# A Randomized, Single-Blind Study Comparing the Clinical Equivalence of Truglyde Fast® and Safil Quick® Polyglycolic Acid Fast-Absorbing Sutures for Episiotomy Repair Following Vaginal Delivery

**DOI:** 10.7759/cureus.42348

**Published:** 2023-07-24

**Authors:** Roopa NK, Geetanjali Devgarha, Y Aruna Subha Shree Rao, Rekha N, Ashok K Moharana, Deepak TS

**Affiliations:** 1 Department of Obstetrics and Gynecology, BGS Global Institute of Medical Sciences and Hospital, Bengaluru, IND; 2 Department of Obstetrics and Gynecology, Marudhar Hospital, Jaipur, IND; 3 Department of Obstetrics and Gynecology, King George Hospital/Andhra Medical College, Visakhapatnam, IND; 4 Department of Clinical Affairs, Healthium Medtech Limited, Bengaluru, IND

**Keywords:** wound complications, vaginal delivery, polyglycolic acid fast absorbable suture, perineal pain, episiotomy

## Abstract

Introduction: Episiotomy, the deliberate surgical incision on the vaginal orifice during vaginal delivery, requires prompt repairing of the incised tissue. It may be associated with bleeding, infection, dehiscence, dyspareunia, short-term pain, and prolonged hospital stay. The outcome of surgery depends on the suture material and technique to repair the episiotomy.

Objective: We aim to subjectively assess perineal pain and maternal morbidity following episiotomy repair with Truglyde Fast^®^ (Healthium Medtech Limited, Bengaluru, India) and Safil Quick^®^ (B. Braun Medical Private Limited, Mumbai, India) polyglycolic acid fast-absorbing suture.

Materials and methods: This multicentric, prospective, randomized (1:1), two-arm, parallel-group, single-blind study was started in August 2020 and completed in March 2021. Ninety-nine primiparous or multiparous eligible women requiring episiotomy were recruited to Truglyde Fast^®^ (n=51) and Safil Quick^®^ (n=48) groups. The primary outcome measure was post-episiotomy perineal pain to be assessed using a visual analog scale (VAS). Secondary endpoints included evaluation of local anesthesia (quantity), intraoperative suture handling, number of sutures utilized, time spent for episiotomy repair and complete healing, analgesic number and dosage, early and late wound complications, presence of residual suture and frequency of re-suturing, resumption of sexual activity and dyspareunia, and adverse events. The threshold to discriminate significant from non-significant outcomes was p<0.05.

Results: At all visits, a non-significant change in perineal pain was noted between Truglyde Fast^®^ and Safil Quick^®^ groups. A significant difference (p<0.05) in the number of sutures used and intraoperative handling characteristics was observed between thegroups. Results of other secondary endpoints showed non-significant differences.

Conclusion: Truglyde Fast^® ^and Safil Quick^® ^polyglycolic acid fast-absorbing sutures are clinically equivalent. Both sutures are safe and effective for episiotomy repair following vaginal delivery with minimal perineal pain and risk of maternal morbidity.

## Introduction

Episiotomy is an incision through the perineum to enlarge the opening of the vagina, facilitate childbirth, and reduce perineal tears [1]. In India, episiotomy is commonly performed during vaginal delivery, with an overall rate of ~70% [2]. It may be associated with bleeding, infection, dehiscence, dyspareunia, short-term pain, and prolonged hospital stay. The long-term effects of surgical repair include anorectal dysfunction, chronic infections, pelvic organ prolapse, and urinary incontinence, among others [3].

Suture material as well as repairing technique influences the outcome of the episiotomy process. The use of absorbable synthetic materials for episiotomy repair is associated with lower short-term morbidity than catgut suture material [4,5]. Polyglycolic acid sutures are synthetic and absorbed by hydrolysis (~42 days), causing less tissue reaction [6]. Although studies are comparing the clinical effectiveness of polyglactin 910 and fast-absorbing polyglactin 910 with catgut [7,8] and fast-absorbing polyglactin 910 sutures (Trusynth Fast^®^ versus Vicryl Rapide^®^) [9] in case of episiotomy repair, no randomized controlled trial was conducted comparing two brands of polyglycolic acid sutures for episiotomy repair till date. Therefore, this study evaluated and compared Truglyde Fast^® ^(Healthium Medtech Limited, Bengaluru, India) and Safil Quick^® ^(B. Braun Medical Private Limited, Mumbai, India) polyglycolic acid sutures for episiotomy repair on maternal morbidity.

## Materials and methods

Study designs

This is a multicentric, prospective, randomized (1:1), single-blind, two-arm, parallel-group study that was started on August 9, 2020, and completed on March 29, 2021. The primary objective of the study was the measurement of perineal pain, occurring after episiotomy repair between Truglyde Fast^®^ and Safil Quick^®^ groups. Secondary objectives were to compare overall intraoperative handling, number of sutures utilized, wound healing, analgesic use, residual suture removal, resumption of sexual activity, dyspareunia, and post-episiotomy maternal morbidities in both groups.

A previous single-blind, randomized study (Clinical Trials Registry - India (CTRI) registration number: CTRI/2020/12/029925) by the same sponsor (Healthium Medtech Limited) has compared the clinical equivalence of Trusynth Fast^®^ and Vicryl Rapide^®^ polyglactin 910 fast-absorbing sutures on maternal morbidity in women undergoing episiotomy repair [9]. The study is similar to the present study, except for the sutures used for episiotomy repair. A likely methodology for conducting research as discussed in the previous study [9] is also followed in the present study.

Ethical approval

The study is prospectively registered with the Clinical Trials Registry - India (CTRI registration number: CTRI/2020/06/026040, date: June 22, 2020) and obtained approval from the Institutional Ethics Committee of each participating site. New Drugs and Clinical Trials Rules 2019, International Council for Harmonisation of Technical Requirements for Pharmaceuticals for Human Use (ICH) Good Clinical Practice E6 (R2) guidelines, European and International Standard (EN ISO) 14155:2020 guidelines, Medical Devices Rules, 2017 (Ministry of Health and Family Welfare), Medical Devices Regulation (EU) 2017/745, and Consolidated Standards of Reporting Trials (CONSORT) were followed.

Participants of the study

Multiparous or primiparous women (aged 18-40 years), having good mental or systemic health and singleton pregnancy with a gestational age of more than 34 weeks, requiring episiotomy during vaginal delivery were included. Informed consent was obtained from all subjects.

The exclusion of participants was based on the presence of perineal/cervical/vaginal tears, an extension of the episiotomy, or intrapartum fever. Moreover, a history of bleeding or coagulation disorder, stillbirth, or perineal surgery (apart from primary repair for childbirth) was the exclusion criterion. Women with hepatitis B infection or human immunodeficiency virus, or allergy to the suture materials, or who were already part of other studies at the same time and had a lower possibility to follow the study procedure were also excluded from the study.

Study settings

Three sites across India were involved: (i) Department of Obstetrics and Gynecology, BGS Global Institute of Medical Sciences and Hospital, Bengaluru, Karnataka, (ii) Marudhar Hospital, Jaipur, Rajasthan, and (iii) Department of Obstetrics and Gynecology, King George Hospital/Andhra Medical College, Visakhapatnam, Andhra Pradesh.

Intervention

Both Truglyde Fast^®^ (Healthium Medtech Limited, Bengaluru, India) and Safil Quick^®^ (B. Braun Medical Private Limited, Mumbai, India) are synthetic absorbable sterile, undyed, surgical sutures, formed of 100% fast absorbable polyglycolic acid. Both Truglyde Fast^®^ and Safil Quick^®^ sutures are intended for the approximation of soft tissue.

Demographics and other characteristics

Ethnicity, age, vital signs, weight and height, parity, gestation period, fetal presentation, history of alcohol consumption, smoking, and previous episiotomy were documented. The medical history of all study participants was also recorded.

Outcomes of the study

Primary Outcome

Perineal pain was measured using a visual analog scale (VAS) at all postpartum follow-ups, where grades were denoted as 0-4 (no pain), 5-44 (mild pain), 45-74 (moderate pain), and 75-100 (severe pain).

Secondary Outcomes

The volume of local anesthesia, intraoperative suture handling, number of sutures, and total time spent on episiotomy repair were measured. Early and late wound complications, as defined by the previous study [9], such as swelling, wound infection, wound dehiscence, hematoma, puerperal fever, urinary incontinence, and frequency of wound re-suturing were also noted. In addition, the number and dosage of analgesics, time taken for complete healing, presence of residual suture, return to sexual activity and dyspareunia, and adverse events were recorded.

Intraoperative suture handling characteristics including ease of passage through tissue, first-throw knot holding, knot tie-down smoothness, knot security, surgical handling, memory, and suture fraying of both sutures were rated as 1 (poor), 2 (fair), 3 (good), 4 (very good), and 5 (excellent). Standardized and valid redness, edema, ecchymosis, discharge, and approximation scoring scale or REEDA (score 0, better healing at the episiotomy site, and score 15, greater tissue trauma) was used for the assessment of wound healing.

Moreover, the duration of the second stage of labor, incision length, number of layers closed, suture-related challenges, perioperative complications, the outcome of surgery, postpartum hemorrhage, hospital stay, the feeling of slight stitches, suture loosening, and if the suture was sent for culture were noted. Prescribed/concomitant medications were also recorded.

Sample size

There is a paucity of data on polyglycolic acid fast sutures in episiotomy repair; hence, the sample size was calculated using data from a study on polyglactin 910 fast-absorbing sutures [7]. According to this study, at six weeks, one out of 50, i.e., 2% of women in the Vicryl Rapide^®^ group, had mild discomfort while sitting, and 98% had no pain or discomfort. Based on the findings, 98% of subjects were assumed to experience no post-episiotomy perineal pain in the standard Safil Quick^®^ arm. Further assuming a 5% type I error, 80% power, and 1% difference in the proportion of subjects having no perineal pain in the Truglyde Fast^®^ arm, with a non-inferior margin of 10% of the difference, the sample size was determined as approximately 38 subjects in each arm. Moreover, the required sample size was increased to 50/arm (total of 100 study participants), after considering 20% dropout and 10% post-randomization exclusion.

Randomization and blinding

A freely available software with block sizes of 4, 6, or 8 was used by an independent programmer to generate three random lists (n=50). Eligible subjects were randomized using sequentially numbered opaque sealed envelopes. Each site followed a competitive enrolment to enroll up to 50 subjects, with 25 subjects randomized to the Truglyde Fast^®^ and 25 to the Safil Quick^®^ suture group, which was stopped when a total of 100 subjects were recruited across three centers. This was a single-blind study; hence, allocation status was not revealed to the subjects.

Procedure for episiotomy repair

At baseline visit (Day 0), the subjects underwent episiotomy, and standard precautions were taken pre-, peri- and postoperatively. Episiotomy was repaired as per standard institutional protocol using either Truglyde Fast^®^ or Safil Quick^®^ suture. Follow-up was done on Day 2, Day 11, Week 6, and Week 12 post-episiotomy.

Statistical analysis

Per-protocol analysis or PP analysis of the study outcomes was carried out with Statistical Package for the Social Sciences (SPSS) version 25.0 (IBM SPSS Statistics, Armonk, NY, USA) that includes all subjects who had complete data for primary outcome during 12 weeks. The Mann-Whitney U test was used to compare differences between distribution-free data, while the t-test was used for normally distributed data, and expressed as mean±standard deviation (SD). Fisher’s exact test or Chi-square test was used to compare qualitative variables, expressed as proportions or percentages. The primary endpoint was summarized as the mean pain score (using the t-test or Mann-Whitney U test) and the proportion of subjects experiencing no perineal pain (using the Chi-square test). Secondary endpoints were expressed as mean±SD or as proportions/percentages based on the quantitative or qualitative nature of the variable. The Mann-Whitney U test was used for additional subgroup analysis. The threshold to discriminate significant from non-significant outcomes was p<0.05.

## Results

**Figure 1:**
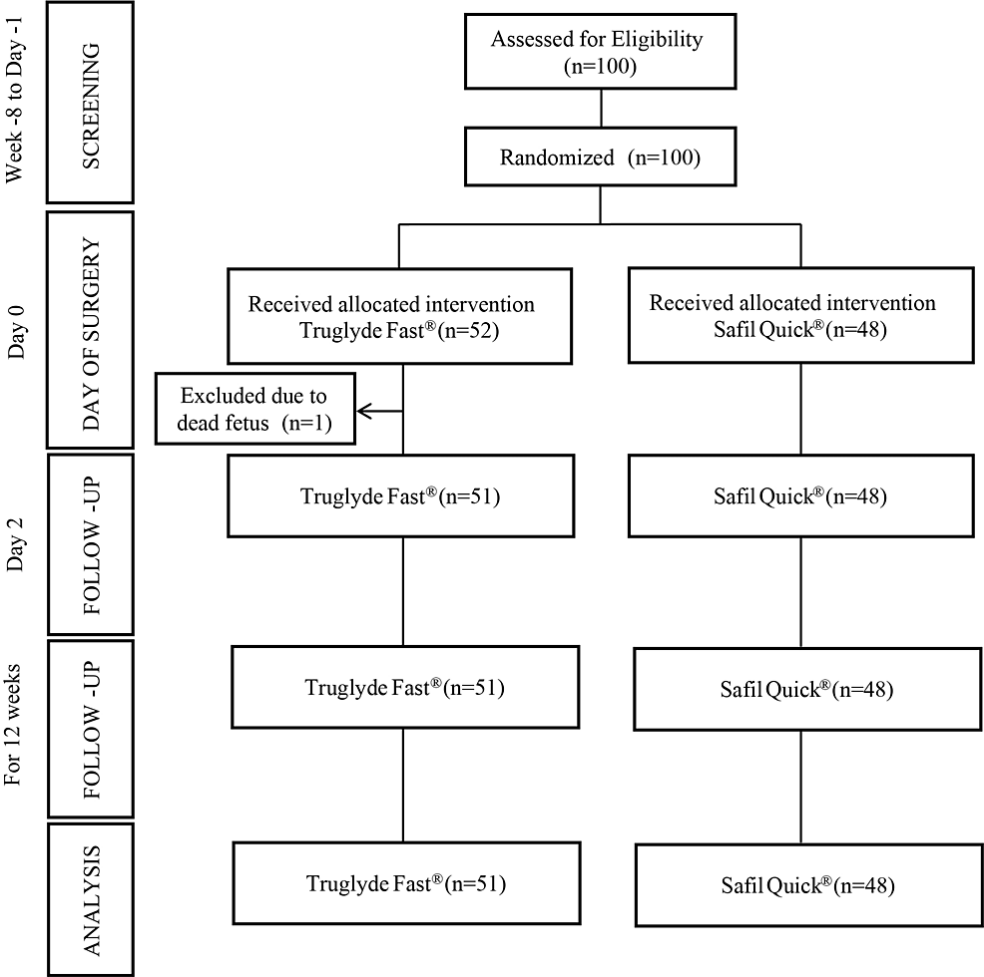
CONSORT flow diagram of participants and the study design CONSORT: Consolidated Standards of Reporting Trials

One hundred women were screened for eligibility and randomized between August 9, 2020, and January 2, 2021, to Truglyde Fast^®^ (n=52) and Safil Quick^®^ (n=48) groups. One subject of the Truglyde Fast^®^ group was excluded after the baseline visit; hence, the PP analysis set comprised 99 subjects, who had complete data on study endpoints until Week 12 (Figure 1).

The follow-up of the last subject was done on March 29, 2021.

Demographics and other characteristics

Indian women with no history of alcohol consumption or smoking were studied. The subject’s demographics and other characteristics are presented in Table 1.

**Table 1:** Demographics and clinical characteristics *p<0.05 BMI: body mass index, SD: standard deviation

Characteristics	Truglyde Fast^®^ (n=51)	Safil Quick^®^ (n=48)	p-value
Age (years), mean±SD	25.60±3.85	24.00±3.70	0.04*
Weight (kg), mean±SD	64.21±9.11	62.89±5.93	0.40
Height (cm), mean±SD	157.23±4.08	157.44±4.59	0.81
BMI (kg/m^2^), mean±SD	25.98±3.57	25.40±2.54	0.36
Pulse rate (beats/minute), mean±SD	83.00±6.38	83.30±7.60	0.83
Respiratory rate (breaths/minute), mean±SD	19.20±2.49	19.40±2.67	0.70
Systolic blood pressure (mmHg), mean±SD	115.08±8.19	115.29±7.61	0.90
Diastolic blood pressure (mmHg), mean±SD	74.40±6.82	74.98±6.75	0.67
Gestation period (weeks), mean±SD	38.11±1.07	38.38±1.19	0.24
Parity number, number (%)			
0	20 (39.22)	27 (56.25)	0.20
1	27 (52.94)	17 (35.42)	
2	3 (5.88)	4 (8.33)	
3	1 (1.96)	0 (0)	
History of previous episiotomy, number (%)	27 (52.94)	16 (33.33)	0.049*
Medical history, number (%)	3 (5.88)	2 (4.17)	0.70

During birth, the fetus of all subjects was in the vertex position.

Analysis of primary outcome

**Figure 2:**
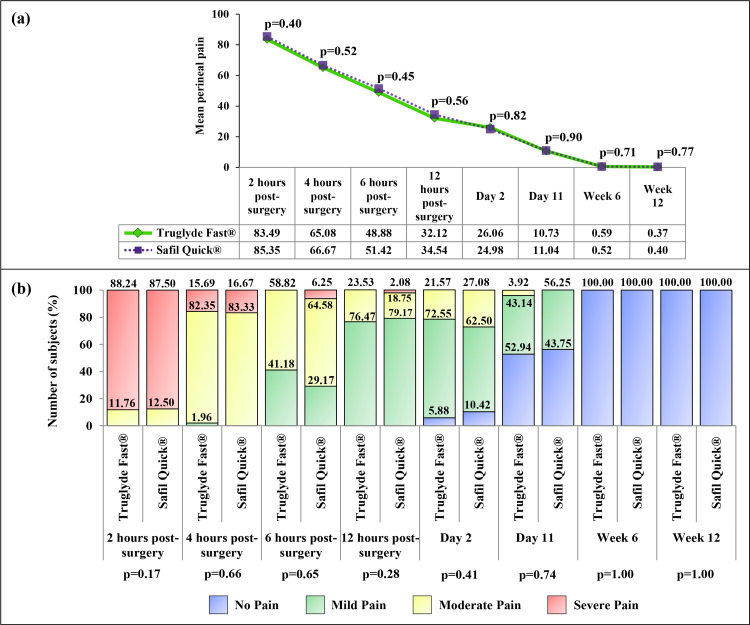
Comparison of postoperative perineal pain between the Truglyde Fast® (n=51) and Safil Quick® (n=48) groups: (a) perineal pain with VAS and (b) different grades of perineal pain (mild, moderate, and severe) VAS: visual analog scale

The mean perineal pain at two hours post-surgery was 83.49 and 85.35, which gradually decreased in intensity with each follow-up and reduced to 0.37 and 0.40 during Week 12, in the Truglyde Fast^®^ and Safil Quick^®^ group, respectively (Figure 2a).

With subsequent follow-up visits, more subjects started experiencing no perineal pain, and none of them had perineal pain by Week 12 (Figure 2b). The results of both mean and grade of perineal pain showed non-significant differences between the groups.

Subgroup Analyses

At baseline, heterogeneity was noted regarding age and history of previous episiotomy between the Truglyde Fast^®^ and Safil Quick^®^ groups. For this reason, subgroup analysis of the primary endpoint was done with respect to age (subgroups: <25 and ≥25 years) and history of previous episiotomy (subgroups: no and yes). No statistical difference was found in mean perineal pain versus age between the groups at any follow-up visits. A statistical difference (p<0.05) in mean perineal pain versus history of previous episiotomy was observed after two hours of surgery on Day 0 that became non-significant in the subsequent visits.

Analysis of secondary outcomes

Intraoperative Profile

Local anesthesia (10 mL lignocaine) has been given to all, except one (1.96%) subject in the Truglyde Fast^®^ group and one (2.08%) subject in the Safil Quick^® ^group, who received 15 mL of lignocaine (p=0.97). All participants had vaginal deliveries, and the instruments used during delivery are summarized in Table 2.

**Table 2:** Intraoperative and postoperative profiles *p<0.05 SD: standard deviation

Characteristics	Truglyde Fast^®^ (n=51)	Safil Quick^®^ (n=48)	p-value
Intraoperative profile			
Quantity of anesthesia (mL), mean±SD	10.10±0.70	10.10±0.72	1.00
Instrumental devices used, number (%)			
No	46 (90.20)	43 (89.58)	0.61
Low outlet forceps	0	1 (2.08)	
Vacuum	5 (9.80)	4 (8.33)	
Duration of the second stage of labor (hours), mean±SD	0.45±0.38	0.52±0.45	0.40
Number of sutures used, mean±SD	1.04±0.20	1.35±0.48	0.0001*
Number of sutures used, number (%)			
1	49 (96.08)	31 (64.58)	0.0001*
2	2 (3.92)	17 (35.42)	
Length of incision (cm), mean±SD	3.05±0.59	3.30±0.85	0.09
Total time for repair of episiotomy (minutes), mean±SD	9.39±3.16	10.85±4.43	0.06
Time of giving episiotomy to time of completion of suturing (minutes), mean±SD	32.16±13.41	32.38±12.71	0.93
Postpartum hemorrhage, number (%)	1 (1.96)	1 (2.08)	0.97
Postoperative profile			
Birth weight of infant, mean±SD	2.86±0.35	2.86±0.41	1.00
Total number of analgesics prescribed, mean±SD	1.87±0.11	1.76±0.10	0.76
Length of hospital stay (days), mean±SD	3.76±1.85	3.58±1.37	0.83
Total score of episiotomy healing scale, mean±SD	0.43±0.23	0.45±0.49	0.79
Swelling, number (%)			
Day 2	23 (45.10)	21 (43.75)	0.96
Day 11	2 (3.92)	4 (8.33)	0.35
Week 6	0 (0)	1 (2.08)	0.29
Week 12	0 (0)	0 (0)	-
Time to complete healing (days), mean±SD	8.86±8.21	10.42±11.66	0.44
Resumption of sexual activity, number (%)			
Week 6	3 (5.88)	5 (10.41)	0.65
Week 12	10 (19.61)	13 (27.08)	0.38

**Figure 3:**
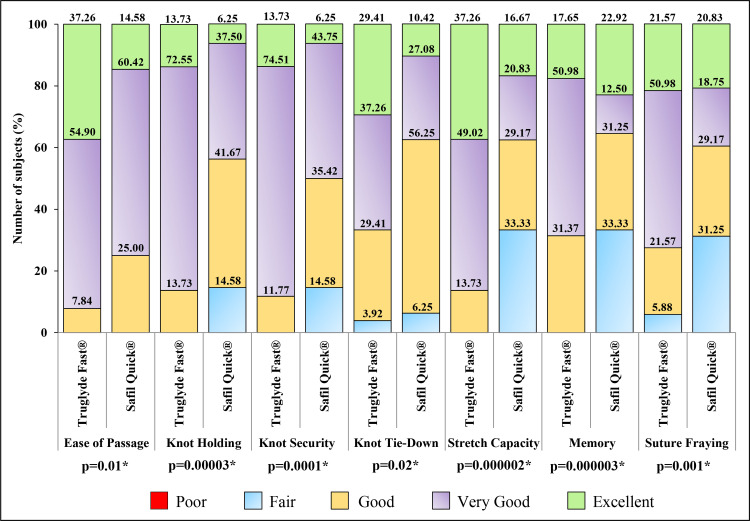
Comparison of intraoperative suture handling characteristics between the Truglyde Fast® (n=51) and Safil Quick® (n=48) groups: representation of percentage scores for grades “excellent,” “very good,” “good,” “fair,” and “poor” *p<0.05

Right mediolateral episiotomy was done to close three layers in all subjects (p=1.00). A significant difference (p<0.05) in intraoperative handling characteristics was noted between the groups (Figure 3).

Both “excellent” and “very good” scores for ease of passage, knot holding, knot security, knot tie-down, stretch capacity, memory, and suture fraying were higher in the Truglyde Fast^®^ than in the Safil Quick^®^ group. In both groups, neither the characteristics were graded “poor” nor any intraoperative suture-related challenges were recorded. No perioperative complications along with the good outcomes of surgery were noted in both groups. Other intraoperative characteristics are shown in Table 2.

Postoperative Profile

**Figure 4:**
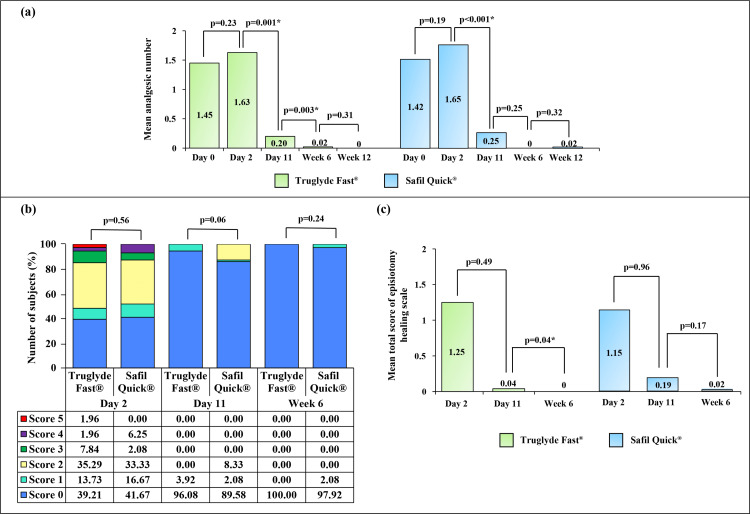
Postoperative profile: (a) change in the mean number of analgesics at each follow-up visit in the Truglyde Fast® (n=51) and Safil Quick® (n=48) groups, (b) comparison of the percentage of subjects with REEDA score at Day 2, Day 11, and Week 6 between the Truglyde Fast® (n=51) and Safil Quick® (n=48) groups (lower score indicated better healing, and higher score indicated tissue trauma), and (c) change in the mean total score of REEDA scale/episiotomy healing scale in the Truglyde Fast® (n=51) and Safil Quick® (n=48) groups at Day 2, Day 11, and Week 6 *p<0.05 REEDA: redness, edema, ecchymosis, discharge, and approximation

The postoperative requirement of analgesics was decreased in both groups with passing visits (Figure 4a).

The overall number of analgesics was comparable between the groups (Table 2). The most common wound complication observed in both groups was swelling, which eventually decreased and became nil by Week 6 in the Truglyde Fast^®^ group and by Week 12 in the Safil Quick^®^ group (Table 2). No other wound complications, viz., hematoma, wound infection, dehiscence or re-suturing, puerperal fever, and urinary incontinence, were observed. Incidence of suture loosening, removal of residual sutures, sent for culture, postoperative suture-related complications, and readmission were not registered. The feeling of slight stitches was noted in subjects of the Truglyde Fast^®^ and Safil Quick^®^ groups on Day 2 (mild: 19.60% versus 16.67%, moderate: 29.41% versus 31.25%, p=0.91) and Day 11 (mild: 31.37% versus 35.42%, moderate: 3.92% vs. 0%, p=0.55). However, during Weeks 6 and 12, none of the subjects in both groups had a feeling of slight stitches.

By Week 6, all subjects of the Truglyde Fast^®^ group showed complete healing in terms of the REEDA scale/episiotomy healing scale (score: 0) (Figure 4b, 4c). The mean score of the episiotomy healing scale was comparable between the groups (Table 2). A similar number of subjects in both Truglyde Fast^®^ and Safil Quick^®^ groups have resumed sexual activity by Weeks 6 and 12 (Table 2), with no incidence of dyspareunia. The overall usage of antibiotics was comparable between the groups. Analgesics, such as paracetamol, diclofenac, aceclofenac, and drotaverine hydrochloride, were prescribed to the majority of the subjects (Table 3).

**Table 3:** Prescribed analgesics during the study

Analgesic medication	Truglyde Fast^®^ (n=51)	Safil Quick^®^ (n=48)
Analgesics, number (%)		
Paracetamol	47 (92.16)	46 (95.83)
Diclofenac	24 (47.06)	24 (50)
Aceclofenac	25 (49.02)	26 (54.17)
Drotaverine hydrochloride	14 (27.45)	17 (35.42)

Six non-serious adverse events occurred during the study period. The events were generally mild and not related to the study device. In the Truglyde Fast^®^ group, one (1.96%) subject had nausea and hypertension, and one (1.96%) subject had abdominal pain. In the Safil Quick^®^ group, one (2.08%) subject reported mild pain in the surgical area, one (2.08%) subject reported general body pains and nausea, one (2.08%) subject reported upper respiratory tract infection, and one (2.08%) subject reported fever and headache.

## Discussion

An episiotomy is a surgical incision through the perineum to widen the vaginal orifice for facilitating the second stage of labor and for promoting maternal and neonatal outcomes [1,10]. Episiotomy is one aspect of childbirth that affects millions of women worldwide by causing perineal pain, physical discomfort, and other complications [11]. Short- and long-term perineal pain and post-delivery complications may be affected by the material of the suture used for episiotomy repair [3,12]. Many studies are available regarding the clinical effectiveness of polyglactin 910 and fast-absorbing polyglactin 910 for episiotomy repair [7-9,13-15], but none compared the two brands of polyglycolic acid sutures. The present study has compared Truglyde Fast^®^ and Safil Quick^®^ polyglycolic acid fast-absorbing sutures in relation to maternal morbidity after episiotomy repair.

The two suture groups showed comparable outcomes with respect to baseline demographics and vital signs, except age and history of previous episiotomy. Although subgroup analysis of the primary endpoint with respect to age was comparable, a significant difference was found with respect to the history of previous episiotomy after two hours of episiotomy repair that became non-significant in the subsequent visits. Many factors including previous episiotomy, use of anesthesia, or the instrument used during delivery can be responsible for this significant difference between the groups in the immediate postpartum period. However, the results of the primary endpoint were not impacted by the heterogeneity, as a non-significant difference in perineal pain was observed at each subsequent visit. A previous study also reported a beneficial effect of rapidly absorbed synthetic suture material regarding less pain and the need for analgesics, compared to catgut [16]. In the present study, the perineal pain became nil at the last two follow-up visits. Additionally, no significant difference in the analgesic requirements as well as a decreasing number of analgesics with each follow-up was found.

A lower number of Truglyde Fast^®^ sutures (p<0.05) were used for episiotomy repair in comparison to Safil Quick^®^ sutures, implicating the economized consumption rate of the Truglyde Fast^®^ suture contrary to the comparator suture. In addition, a significant difference was observed in intraoperative suture handling parameters between the groups. A comparatively higher “excellent” and “very good” scores for all of the handling characteristics in the Truglyde Fast^®^ group demonstrated better overall intraoperative suture handling with Truglyde Fast^®^ suture than with Safil Quick^®^. Other intraoperative characteristics were comparable between the groups. Good outcomes of surgery in both groups indicated clinical equivalence of Truglyde Fast^®^ and Safil Quick^®^ suture use.

Although episiotomy has maternal benefits, still, postpartum infections within six weeks of delivery are accountable for maternal morbidity and mortality. This increases socioeconomic and healthcare burdens as well as maternal anxiety and depression that negatively impact newborn care [17]. In the present study, none of the subjects reported wound infection, wound dehiscence, hematoma, and puerperal fever. However, the incidence of swelling was observed to start to decrease on Day 11 and became nil by Week 6 in the Truglyde Fast^®^ group and by Week 12 in the Safil Quick^®^ group. Due to their fast absorbability, suture removal was not required in any studied group. The groups were not significantly different regarding the feeling of slight stitches on Day 2 and 11 post-episiotomy. Episiotomy is considered to heal rapidly, consequently lowering the risk of sexual dysfunction and urinary and/or fecal incontinence [3]. However, postpartum wound infection can cause long-term dyspareunia [11]. The subjects of the present study reported no incidence of urinary incontinence. Even some of them have resumed their sexual activity with no complaint of dyspareunia. The REEDA scale is a tool for assessing inflammation induced by episiotomy; signs include redness, edema, ecchymosis, discharge, and apposition [18]. The mean REEDA score improved with passing visits in both groups of this study.

The limitation of the study was that only subjects were blinded to the intervention used, as this was a single-blind study, but potential bias may have occurred regarding the assessment of intraoperative handling, if surgeons favored one suture over the other. However, the study design is methodologically robust, and other objective assessments and clinical endpoints during the follow-up could not be influenced by this bias. Safety and efficacy analyses of Truglyde Fast^®^ polyglycolic acid fast-absorbing suture further confirmed that it can be generalized to a wider population and can be used for all surgeries indicated for Safil Quick^®^ polyglycolic acid fast-absorbing suture.

## Conclusions

Our study revealed that Truglyde Fast^®^ polyglycolic acid fast-absorbing suture is clinically equivalent to the Safil Quick^®^ polyglycolic acid fast-absorbing suture as non-significant differences regarding both primary and secondary endpoints (except the number of sutures used and intraoperative suture handling) were recorded. Therefore, following vaginal delivery, an episiotomy can be repaired safely and effectively using both Truglyde Fast^®^ and Safil Quick^®^ sutures, with minimal perineal pain and a low risk of maternal morbidity.
